# Use of Multispectral Imaging in Varietal Identification of Tomato

**DOI:** 10.3390/s150204496

**Published:** 2015-02-16

**Authors:** Santosh Shrestha, Lise Christina Deleuran, Merete Halkjær Olesen, René Gislum

**Affiliations:** Department of Agroecology, Faculty of Science and Technology, Aarhus University, Slagelse 4200, Denmark; E-Mails: santosh.shrestha@agro.au.dk (S.S.); lise.deleuran@agro.au.dk (L.C.D.) merete.olesen@agro.au.dk (M.H.O.)

**Keywords:** tomato, seed, varietal discrimination, varietal identification, multispectral imaging, non-destructive

## Abstract

Multispectral imaging is an emerging non-destructive technology. In this work its potential for varietal discrimination and identification of tomato cultivars of Nepal was investigated. Two sample sets were used for the study, one with two parents and their crosses and other with eleven cultivars to study parents and offspring relationship and varietal identification respectively. Normalized canonical discriminant analysis (nCDA) and principal component analysis (PCA) were used to analyze and compare the results for parents and offspring study. Both the results showed clear discrimination of parents and offspring. nCDA was also used for pairwise discrimination of the eleven cultivars, which correctly discriminated upto 100% and only few pairs below 85%. Partial least square discriminant analysis (PLS-DA) was further used to classify all the cultivars. The model displayed an overall classification accuracy of 82%, which was further improved to 96% and 86% with stepwise PLS-DA models on high (seven) and poor (four) sensitivity cultivars, respectively. The stepwise PLS-DA models had satisfactory classification errors for cross-validation and prediction 7% and 7%, respectively. The results obtained provide an opportunity of using multispectral imaging technology as a primary tool in a scientific community for identification/discrimination of plant varieties in regard to genetic purity and plant variety protection/registration.

## 1. Introduction

Tomato (*Solanumlycopersicum* L.) is one of the leading horticultural crops in the world and number one vegetable in terms of economic value generated [1]. Intense increase in demand due to its diverse human consumption (salad, ketchup, paste, powder, *etc.*) and health benefits (vitamin C, lycopene and β-carotene) has led to intensive breeding efforts. This has resulted in development and release of a large number of diverse tomato cultivars commercially available in the market. The identification and discrimination of these cultivars is vital at all stages of the seed production chain. The importance lies with equal magnitude for all stakeholders involved in crop production, *i.e.*, breeders, seed producers, processors, seed traders, variety registration and certification agencies and other end-users in terms of requirement and benefits obtained from it [2]. The growing seed business at international and national level has created an interest in descriptive characterization of the plant varieties in the context of intellectual property protection, a compliance to agreement within the framework of the World Trade Organization (WTO) [3]. Nepal being a member of WTO has an obligation to fulfill the requirements in the WTO Trade-related Aspects of Intellectual Property Rights (TRIPS) agreement, and has drafted a bill called “Plant Variety Protection and Farmer's Rights” (under consideration) *sui generis* of TRIPS and has emphasized plant breeders rights.

Morphological characters are distinct and stable and often used for identification of the varieties. However, intensive modern breeding technology has created a narrow genetic diversity resulting in lack of minimum phenotypic variation among the germplasm making morphological markers insufficient and extremely difficult to use for identification. Several methods such as biochemical (protein) markers [4,5] and molecular markers [6–10] have been investigated and developed for robust identification and characterization of tomato germplasm. However, these methods are costly, destructive and time consuming, and to overcome these shortcomings and to meet the modern crop production demands, technologies which are quick and reliable in identifying the tomato varieties for technical and economic aspects are desirable and beneficial [11].

Multispectral imaging is a developing non-destructive technology, which combines the benefits of conventional imaging and spectroscopy technique by attaining both spatial and spectral information from the object simultaneously. Analyses from multispectral imaging are well suited for on-line process monitoring and quality control as they are non-destructive, simple and rapid does not require sample pre-treatments. This technology also presents an opportunity to measure different components at the same time for quality assurance [12,13]. Multispectral imaging has been used to predict unripe tomatoes with an accuracy of 85% [14], bioactive compounds in intact tomato fruit [12] and has also been used for identification of cherry-tomato with a prediction accuracy of 80% [15]. Further, it has also been reported to discriminate between transgenic and non-transgenic rice [13]. Similar non-destructive technology like hyperspectral imaging had been used in discrimination of maize varieties [16] and wheat classes [17]. However, to our knowledge, there are no published data on multispectral imaging or any other similar technologies for variety identification of tomato using the individual seeds.

Thus, multispectral imaging technique is proposed and investigated for tomato variety identification. The paper aims to assess the potentiality of multispectral image analysis for classifying and identifying tomato cultivars from Nepal. The study also examines the relationship/hybridity of parent and offspring.

## 2. Experimental Section

### 2.1. Tomato Seed Samples

Eleven tomato cultivars/accessions were collected from different seed agencies in Nepal (Table 1). Pertaining to their differences in cultivation practices and environmental condition and time of production, these tomatoes were grown in 10-litre pots with standard recommended fertilizers application at semi-field conditions in 2014 at Flakkebjerg (Slagelse, Denmark) to reduce any variation resulting from seasonal or growing conditions. Tomatoes were harvested at red ripe stage and seeds were extracted by natural fermentation process (pulp with seeds were collected and allowed to stay overnight and later washed to extract seeds) for each cultivar. The extracted seeds were allowed to dry for two days at room temperature and further dried for three days with fan. These seeds were stored at six degree Celsius (6 °C) until further study. The study was performed with two sample sets. The first set comprised two cultivars- HRD 1 and HRD 17 and their two crosses (HRD 1 × HRD 17 and HRD 17 × HRD 1) for studying parent and offspring relationship and the second sample set comprised of all eleven cultivars (Table 1) to assess the potentiality of multispectral imaging for varietal discrimination.

### 2.2. Image Acquisition and Analysis

#### 2.2.1. Image Acquisition and Pre-Processing

**Figure 1.**
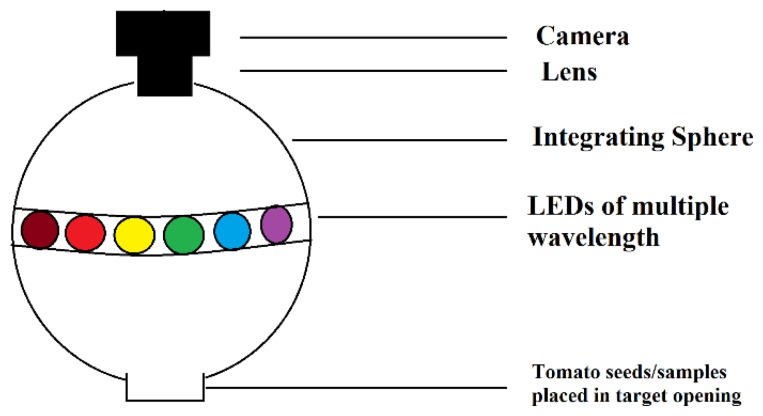
VideometerLab instrument structural set up for capturing multispectral images.

Images from each seed sample were captured using a VideometerLab instrument (Videometer A/S Hørsholm, Denmark, Figure 1). This instrument acquires multispectral images in 19 wavelengths (375, 405, 435, 450, 470, 505, 525, 570, 590, 630, 645, 660, 700, 780, 850, 870, 890, 940 and 970 nm). The instrument consists of a sphere, which is coated with matte titanium paint, and it ensures that light is scattered evenly around the object. The 19 light emitting diodes (LED) are placed along the rim of the sphere and a camera is top-mounted. Before images were captured the instrument was calibrated in respect to color, geometry and self-illumination to ensure a set of direct comparable images.

The images include information of both the seeds, which is the region of interest, and the background (blue-plate), which is not relevant for the analysis. So, to ensure that this irrelevant information does not interfere with the analysis, a pre-determined mask that removes blue background was employed.

#### 2.2.2. Data Analysis

After capturing the images data extraction and transformation of pixel data were done in the VideometerLab software (version 2.13.83). Different algorithms were used for the data analysis and are summarized in Table 2 and are explained below.

Normalized canonical discriminant analyses (nCDA) were used for discrimination between the cultivars. The nCDA is a supervised model based on multispectral imaging (MSI) transformation of the images, in order to minimize the distance to observations within classes and to maximize the distance to observations between classes [18]. Several nCDA transformations were used for the analyses, one of which comprised all cultivars and several other pairwise nCDA MSI transformations.

All seed images were collected into a blob database where each blob was a representation of one seed. Different features from blob toolbox were extracted and calculated for discrimination of tomato cultivars. The features included shape features; area, length, width, roundness and color features; intensity (mean pixel intensity of the image), hue, saturation, CIELab L *, CIELab a *, CIELab b * and RegionMSImean of the blob toolbox which were taken as variables for varietal discrimination.

RegionMSImean calculates a trimmed mean of nCDA-transformed pixel values within the blob. Different RegionMSImean were calculated, one with nCDA MSI transformation of all cultivars or parents and offspring in case of hybridity/relationship study was regarded as “MSI universal” and others with their specific pairwise MSI transformations. Different threshold values were selected for classifying a different pair of cultivars, depending on the selection of MSI transformation used for achieving the best separation between each respective pair of cultivars. These threshold values were later tested on unknown set of seeds for prediction. The RegionMSImean values along with seed shape, color and texture pixel values generated from the blob database were collected into an Excel sheet for further data analysis in the MATLAB software version 8.1.0.604 (R2013a) (The Math Works, Inc., Natick, MA, USA) along with the PLS toolbox 7.9 (Eigenvector Research, Inc., Wenatchee, WA, USA).

Principal component analysis (PCA) [19] was used to explore and compare results of the RegionMSImean discrimination and dataset generated from blob database. The data consisted of the pixel values extracted from blob toolbox for four RegionMSImean values (of parents and offspring, parents only, offspring only, cross HRD 17 with (HRD 17 × HRD 1) and MSI universal), intensity, hue, saturation, CIELab L *, CIELab a *, CIELab b * along with shape features (area, width, roundness and length). Data were auto-scaled before analysis. The optimum numbers of principal components (PC) were chosen at the point where the root mean square of cross validation (RMSECV) was lowest. It was used to analyze and explore the data variability on sample set of parent and offspring discrimination, only.

PLS discriminant analysis (PLS-DA) [20] was used as a supervised multivariate technique for discrimination of the cultivars from the dataset obtained from the blob database which consisted extracted pixel values of blob features of shape and color. There were total of 49 variables out of which 38 were RegionMSImean of 38 different pairwise nCDA MSI transformation of tomato cultivars, one RegionMSImean of nCDA MSI transformation of all cultivars (MSI universal) and the rest were other color and shape features as similar to PCA. For analysis data were first arranged in a 2-D matrix (X) where the rows represent the values obtained from different blob features (color, shape and texture features). One column vector (Y) containing the dependent variable (cultivar category) was assigned to the matrix. The data were auto-scaled before analysis and were cross-validated with random subsets of 15 splits and 20 iterations. The optimum number of latent variables (LVs) was determined by classification error for cross validation and RMSECV to avoid over-fitting of the data. The classification accuracy of the PLS-DA was determined by the number of correctly classified seed samples per cultivar divided by the total number of samples in the class (sensitivity, %). The overall correct classification (accuracy, %) of the model was also calculated as the number of correct classifications in all classes divided by the total number of seed samples analyzed [21]. The developed PLS-DA model was used to predict the unknown set of samples from data which were obtained from the blob database.

## 3. Results and Discussion

### 3.1. Hybridity Study on Tomato

Two different approaches normalized canonical discriminant analysis (nCDA) discrimination and PCA on datasets extracted from the blob database were used to analyze and explore the possibility of using multispectral imaging as a tool for discrimination of parents and their offspring. nCDA was used in these two approaches as the data generated were function of nCDA MSI transformation on seed images. Apart from the nCDA transformations, PCA data also included other blob features pixel values of shape and color.

**Figure 2.**
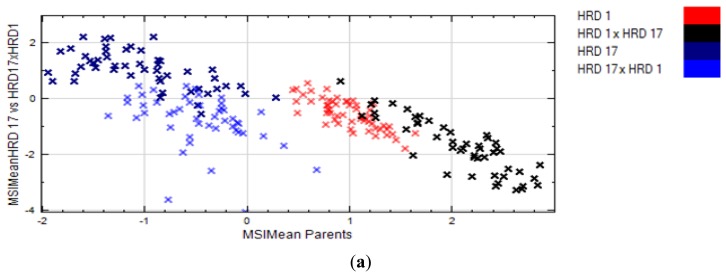

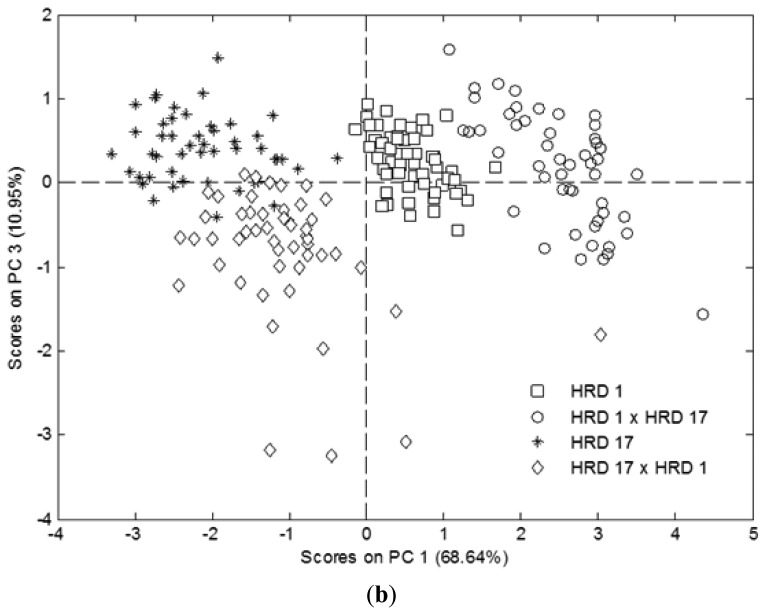
Discrimination of parent and their crosses by (**a**) nCDA; (**b**) PCA on blob dataset.

**Figure 3.**
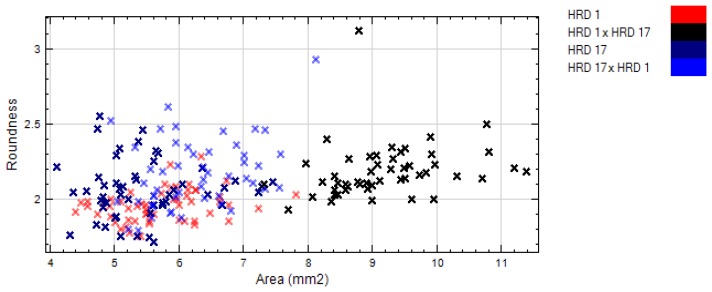
nCDA discrimination on seed shape feature (area *vs.* roundness).

RegionMSImean was able to discriminate parents and their crosses. nCDA transformation of the two parents and of HRD 17 and its offspring (HRD 17 × HRD 1) gave the best separation (Figure 2a). Shape features like area, length, *etc.* of seeds were also analyzed but displayed inferior separation, except for the cross HRD 1 × HRD 17, which showed good separation with others (Figure 3).

A clear separation between parents and the crosses were also found on PCA, which contained the same information of RegionMSImean values along with shape and color pixel values of seeds extracted from the blob database (Figure 2b). The PCA model explained 99.6% of variation in the dataset and Principal Component 1 (68.6% of variation explained) contained the major information for separation between the parents and offspring whereas Principal Component 3 (10.9% of variation explained) separated HRD 17 and its offspring (HRD 17 × HRD 1).

**Figure 4.**
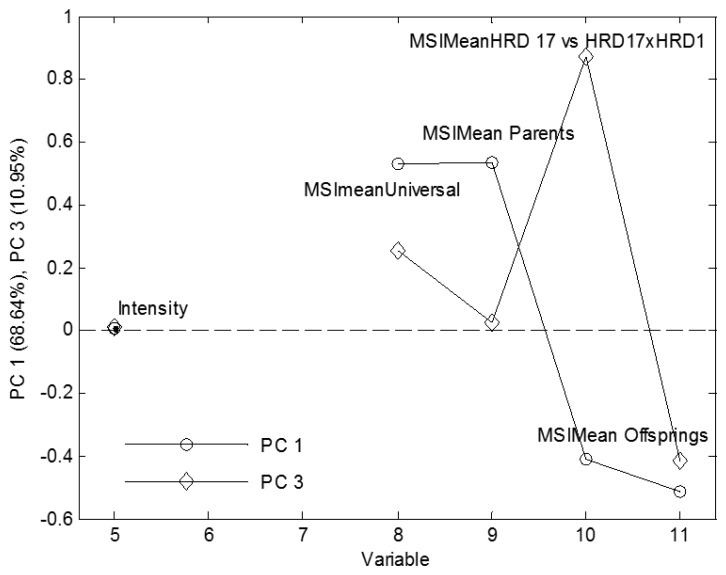
Loading plot obtained from PCA showing factors important for discrimination.

The loading plot (Figure 4) shows the same information responsible for separation as obtained from nCDA discrimination, *i.e.*, nCDA transformation on two parents and other transformation comprising HRD 17 and its offspring (HRD 17 × HRD 1).

HRD 1 × HRD 17 indicated a positive heterosis in terms of increased seed size while the other cross did not show any apparent heterosis. The increase in seed size has been correlated with increased seed storage reserves which could be further linked with seed vigor and robust plant establishment. Studies in spinach suggest that the larger seeds produce vigorous plants [22,23] and often improve seed yield in the subsequent generation [24]. The maternal effect on the seed traits can be observed on the offspring by their affinity towards their respective female parent. The effect is more prominent with HRD 17 and its offspring as it could not be distinctly separated from each other. The seeds are borne on the fruits of female parent and so they have the more possibility of acquiring maternal traits during seed development as compared to male parent [25].

The results obtained by the nCDA discrimination and PCA on datasets extracted from the blob database are comparable with each other on separation, though separation is more distinct on PCA as compared to the other. The other features on seed shape and color which were also included in the analysis seem to have a positive effect on discrimination. So, it is advisable to use others features along with nCDA MSI transformation for better results. The results also suggest that data generated from the blob toolbox, which included nCDA transformation and simple features on shape and texture, can be used for other mathematical calculations and interpretations of data.

### 3.2. Varietal Discrimination

#### 3.2.1. VideometerLab Software Analysis (nCDA)

**Figure 5.**
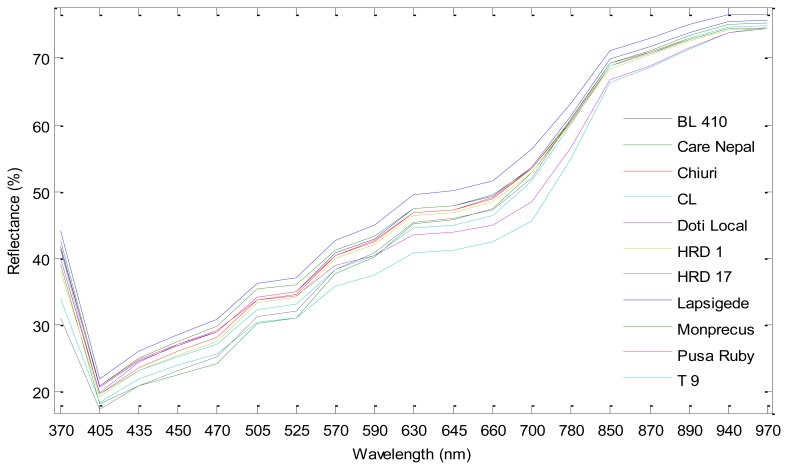
Mean spectrum of eleven cultivars.

**Figure 6.**
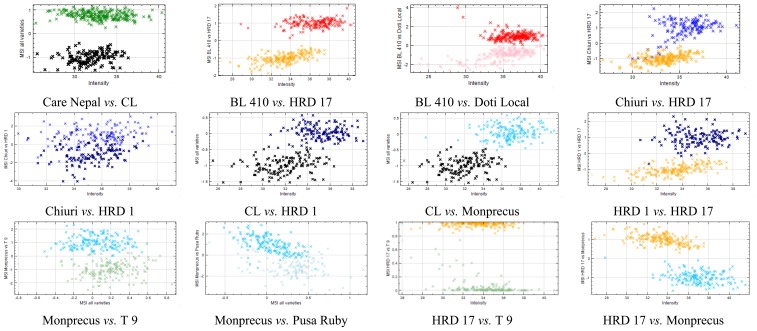
nCDA pairwise discrimination of randomly selected cultivars.

All eleven cultivars have variation among them as depicted by the mean pixel intensity of each cultivar (Figure 5). Pairwise comparisons were done with all cultivars to assess the diversity among cultivars and discriminatory ability of multispectral imaging using VideometerLab software. The several features (color/shape) were tested against each other to find the best separation between cultivars. Shape features like area, length, width, *etc.*, which represent the pixel area/size of the seed images, did not give a good separation for any cultivars, suggesting that the physical parameter of tomato seed shapes are more or less similar to each other, and therefore an insufficient trait for varietal identification. Gunn and Gaffney [26] also reported that the external seed characters (seed size and shape) are not always enough for reliable seed identification. Blob feature RegionMSImean when compared with intensity (mean pixel intensity of the image) gave best separation. MSI universal (RegionMSImean comprising nCDA transformation of all cultivars) was able to discriminate between some pairs of cultivars, mostly those involving Care Nepal and CL. For the rest of the pairs, MSI local (RegionMSImean comprising nCDA transformation between two respective cultivars) gave the best separation (Figure 6). Threshold values were determined as zero for pairs involving MSI local (involving two selected varieties) and a value for MSI universal (includes all varieties for a transformation) for best separation between the pairs and were later used for validation on unknown set.

Pairwise comparisons clearly indicated the presence of diversity among the cultivars as most of the pairs were separable with more than 95% sensitivity (accuracy between selected two cultivars) (Table 3a). Some pairs had sensitivity of 100% whereas very few pairs of cultivars had sensitivity less than 85%. The validations on the unknown set results are very much comparable with calibration set (Table 3b) in terms of robustness of the model to correctly discriminate between the pairs.

The tomato cultivars Chiuri and Pusa Ruby seem to have the similar seed traits (color/shape) to all other cultivars as their discrimination to other selected cultivars had least sensitivity as compared to the other pairs of cultivars which do not involve these two cultivars. The analysis on tomato parents and offspring confirms that the data generated could be explored in various analyses for extrapolation of the data. The generated datasets were used for classifying and validating an unknown set of eleven tomato cultivars using PLS-DA.

#### 3.2.2. PLS-Discriminant Analysis (PLS-DA)

**Figure 7.**
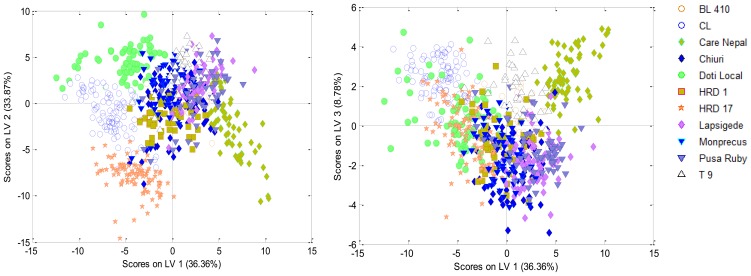
Score plot showing the clustering of eleven tomato cultivars.

A distinct separation between all eleven cultivars could not be observed on the score plot (Figure 7) of PLS-DA (Model A), though some cultivar's clusters were discretely identifiable in the score plot whereas few other cultivars were grouped into one cluster. The developed PLS-DA model (Model A) had an overall accuracy of 82%, 81% and 79% for calibration, cross-validation and prediction set (Table 4) respectively. It was able to separate HRD 17, CL, Care Nepal, T 9 and Doti Local with more than 90% accuracy, as expected from the score plot. However, some of the cultivars could not be separated, e.g., Chiuri from Pusa Ruby and Lapsigede from Monprecus. nCDA discrimination studies also showed that Chiuri and Pusa Ruby were poorly discriminated. Subsequently, stepwise PLS-DA models were developed. Model B was developed based on HRD 17, CL, Care Nepal, Doti Local and T9. Model C consisted of data from the cultivars that could not be separated (Chiuri, Lapsigede, Monprecus and Pusa Ruby). These stepwise classifications increased the overall accuracy of the model from 90% and 66% to 93% and 86% for Model B and C respectively (Table 4). The sensitivity for cultivar classification increased for the poorly classified cultivars from 54%, 58%, 74%, and 74% to 83%, 77%, 92%, and 91% for Chiuri, Pusa Ruby, Lapsigede and Monprecus respectively. The stepwise classifications also increased the sensitivity for BL 410 and HRD 1 from 82% and 77% to 91% and 85% respectively. The averaged classification errors for cultivars decreased when stepwise models were developed reducing from 9% to 7% and were stable in cross-validation and prediction sets (Table 5), though averaged root mean square for calibration/cross validation/prediction increased from 22%/22%/22% to 24%/25%/25% (Table 5) respectively. The decrease in classification error could be well perceived with increase in sensitivity for all cultivars. PLS-DA models contained the variables which were best identified RegionMSImean values for discrimination between either two cultivars along with pixel values of shape and color features which did not contribute much to separation and this is observable in terms of RMSEC/CV/Pred. These features were purposefully included in the dataset, though they were not found to have distinct role in discrimination. However, their cumulative effect could play a positive part in increased sensitivity for classification in models.

Similar studies on varietal identification on tomato have also been successfully demonstrated using another non-destructive technology. VIS-NIR spectroscopy was used to classify tomato plants using top-canopy leaves to classify two tomato varieties [27] and tomato fruits to discriminate/classify transgenic and non-transgenic plants [11,28]. However, this is the first report on use of single seeds for varietal identification on tomatoes.

As morphological markers are incompetent in assessing differences of genetic identities and relationships among varieties having modifications on quality traits (seed or useful economic attributes) and as application of molecular markers requires high cost, time and is sophisticated [29], multispectral imaging provides a wider opportunity to integrate at commercial and scientific level. Its proven capacity to separate the individual with high genetic similarity like transgenic crops [13] gives further significance on its endorsement at any level.

## 4. Conclusions

The study shows the potentiality of using multispectral imaging for studying the parents and offspring relationship and rapid varietal identification of different tomato cultivars. Further, the multispectral imaging gives an advantage of acquiring spectral information (VIS-NIR), which could further be correlated to a specific functional group for biochemical interpretation. It gives an opportunity for mainstreaming online sorting of seeds of different varieties of tomatoes with higher precision whenever conflicts arise on the genetic purity of the seed lot. The multispectral imaging could also be used as a pre-screening technique for identifying and classifying breeding materials along with a diversity study of germplasm as it provides opportunity to include physical traits (seed shape, visual inspection, *i.e.*, RGB) and chemical information (NIR region). Further studies will explore its applicability on varietal discrimination using the spectral information (VIS-NIR) obtained from multispectral imaging and relationship with functional biochemical on tomato and other crops.

## Figures and Tables

**Table 1.** Details of tomato sets (cultivar/accession, number of seeds, seed source and remarks) used in this study.

**Cultivar/Accession**	**Number of Seed Used**			**Seed Source**	**Remarks**

	**Calibration**	**Prediction**	**Total**		
**Sample set One**					

HRD 1	55	-	55	NARC, Nepal	Breeding Material
HRD 17	50	-	50	NARC, Nepal	Breeding Material
HRD 1 × HRD 17	50	-	50	Crossed at Semi-field	HRD 1 as female parent
HRD 17 × HRD 1	50	-	50	Crossed at Semi-field	HRD 17 as female parent
**Sample set Two**					

BL 410	176	50	226	SEAN Seed, Nepal	Released Cultivar
Care Nepal	225	66	291	Seed retailer, Nepal	Farmer's variety
Chiuri	133	76	209	Seed retailer, Nepal	Farmer's variety
CL (also known as NCL)	134	95	229	SEAN Seed, Nepal	Released Cultivar
Doti Local	171	65	236	SEAN Seed, Nepal	Farmer's variety
HRD 1	134	54	188	NARC, Nepal	Breeding Material
HRD 17	192	91	283	NARC, Nepal	Breeding Material
Lapsigede	172	71	243	SEAN seed, Nepal	Released Cultivar
Monprecus	160	58	218	VDD, Nepal	Released Cultivar
Pusa Ruby	137	59	196	NARC, Nepal	Released Cultivar
T 9	169	37	206	SEAN Seed, Nepal	Breeding Material

**Table 2.** Overview of data analysis.

**Algorithm Used**	**Sample Set**	**Variables Used**	**Remarks**
nCDA discrimination	One	RegionMSImean calculated on nCDA MSI transformation of all (parents and offspring) and pairwise transformation of parents, intensity (mean pixel intensity of the image) and offspring along with shape feature viz., area, length, roundness and width.	Hybridity/relationship of parents and offspring.

	Two	RegionMSImean calculated on nCDA MSI transformation of all cultivars and pairwise MSI transformations between two cultivars intensity (mean pixel intensity of the image).	Pairwise comparison between all cultivars.

PCA	One	RegionMSImean values calculated on nCDA MSI transformation including all (parents and hybrids), intensity (mean pixel intensity of the image) and other features on shape and color values extracted from blob database.	Hybridity/relationship of parents and offspring.

PLS-DA	Two	RegionMSImean values calculated on nCDA MSI transformation one including all cultivars and several other pairwise MSI transformations along with shape feature viz., area, roundness, length and width and color features like CIELab L *, CIELab a *, CIELab b *, intensity (mean pixel intensity of the image), saturation, and hue values extracted from blob database.	Classification/identification of tomato cultivars.PLS-DA model containing all eleven varieties (Model A) was developed and further two stepwise models (Model B and Model C) were developed to improve the accuracy of all cultivars.

**Table 3.** Pairwise sensitivity of nCDA discrimination of Tomato cultivars (sensitivity- number of correctly classified seed samples in cultivar divided by the total number of samples in the class).

**A. Calibration Results**										

	**BL 410**	**Care Nepal**	**Chiuri**	**CL**	**Doti Local**	**HRD 1**	**HRD 17**	**Lapsigede**	**Monprecus**	**Pusa Ruby**
Care Nepal	96%									
Chiuri	90%	94%								
CL	99%	100%	98%							
Doti Local	100%	100%	99%	96%						
HRD 1	94%	98%	84%	99%	100%					
HRD 17	100%	100%	97%	98%	100%	99%				
Lapsigede	94%	97%	89%	99%	100%	92%	98%			
Monprecus	98%	99%	84%	99%	96%	89%	99%	95%		
Pusa Ruby	89%	96%	81%	99%	93%	88%	99%	83%	88%	
T 9	97%	96%	94%	99%	93%	96%	100%	94%	96%	92%

**B. Prediction Results**										

	**BL 410**	**Care Nepal**	**Chiuri**	**CL**	**Doti Local**	**HRD 1**	**HRD 17**	**Lapsigede**	**Monprecus**	**Pusa Ruby**
Care Nepal	97%									
Chiuri	78%	94%								
CL	100%	99%	97%							
Doti Local	98%	99%	98%	98%						
HRD 1	91%	98%	72%	98%	100%					
HRD 17	99%	99%	97%	96%	100%	100%				
Lapsigede	93%	99%	81%	99%	99%	92%	98%			
Monprecus	99%	99%	77%	96%	93%	86%	99%	95%		
Pusa Ruby	88%	99%	85%	95%	100%	93%	99%	86%	91%	
T 9	99%	98%	91%	95%	88%	96%	100%	93%	94%	89%

**Table 4.** PLSDA classification of eleven tomato cultivars—Model A) includes all the cultivars, stepwise PLS-DA classification—Model B) includes cultivars with higher sensitivity and Model C) includes cultivars with poor sensitivity from Model A. Previous Overall accuracy (OA) was calculated using the number of correct classifications in selected classes divided by the total number of seed samples of selected classes of Model A.

**Model A**				**Model B**				**Model C**			
		
**Cultivar**	**Calibration**	**CV**	**Prediction**	**Cultivar**	**Calibration**	**CV**	**Prediction**	**Cultivar**	**Calibration**	**CV**	**Prediction**
BL 410	82%	82%	90%	BL 410	91%	90%	98%	Chiuri	83%	81%	78%

CL	94%	94%	95%	CL	96%	96%	97%	Lapsigede	92%	91%	89%

Care Nepal	92%	92%	97%	Care Nepal	92%	92%	97%	Monprecus	91%	89%	91%

Chiuri	54%	53%	39%	Doti Local	92%	91%	91%	Pusa Ruby	77%	75%	80%

Doti Local	91%	91%	95%	HRD 1	85%	85%	87%	**Overall Accuracy**	**86%**	**85%**	**84%**

HRD 1	77%	76%	80%	HRD 17	99%	99%	100%	**Previous (OA)**	**66%**	**65%**	**54%**

HRD 17	98%	98%	98%	T 9	93%	92%	97%				

Lapsigede	74%	72%	70%	**Overall Accuracy (OA)**	**93%**	**92%**	**96%**				

Monprecus	74%	73%	57%	**Previous (OA)**	**90%**	**90%**	**94%**				

Pusa Ruby	58%	58%	49%								

T 9	91%	91%	97%								

**Overall Accuracy**	**82%**	**81%**	**79%**								

**Table 5.** Average classification error of PLSDA models.

**PLS-DA Models**	**Class Err (Cal)**	**Class Err (CV)**	**Class Err (Pred)**	**RMSEC**	**RMSECV**	**RMSEP**
Model A	0.09	0.09	0.10	0.22	0.22	0.22
Stepwise PLSDA (Model B and Model C)	0.07	0.07	0.07	0.24	0.25	0.25
